# Assessment of new hydrogen peroxide activators in water and comparison of their active species toward contaminants of emerging concern

**DOI:** 10.1038/s41598-024-59381-0

**Published:** 2024-04-23

**Authors:** Giulio Farinelli, Jean-Noël Rebilly, Frédéric Banse, Marc Cretin, Damien Quemener

**Affiliations:** 1 Institut Européen des Membranes, IEM-UMR 5635, ENSCM, CNRS, Univeristé de Montpellier, 34090 Montpellier, France; 2 Institut de Chimie Moléculaire et des Matériaux d’Orsay (ICMMO), CNRS, Université Paris-Saclay, 91400 Orsay, France

**Keywords:** Bioinspired complexes, Advanced oxidation processes, Contaminants of emerging concern, Hydroxyl radical, Metal-based pathway, Environmental sciences, Pollution remediation

## Abstract

Advanced oxidation processes are the most efficient tool to thwart the overaccumulation of harmful organic compounds in the environment. In this direction bioinspired metal complexes may be a viable solution for oxidative degradations in water. However, their synthesis is often elaborated and their scalability consequently low. This study presents alternative easy-to-synthesize bioinspired metal complexes to promote degradations in water. The metals employed were iron and manganese ions, hence cheap and highly accessible ions. The complexes were tested toward Phenol, Estrone, Triclosan, Oxybenzone, Diclofenac, Carbamazepine, Erythromycin, Aspartame, Acesulfame K, Anisole and 2,4-Dinitrotoluene. The reaction favoured electron-rich compounds reaching a removal efficiency of over 90%. The central ion plays a crucial role. Specifically, Mn(II) induces a non-radical pathway while iron ions a predominant radical one (^⋅^OH is predominant). The iron systems resulted more versatile toward contaminants, while the manganese ones showed a higher turn-over number, hence higher catalytic behaviour.

## Introduction

Water is overall a fragile matrix: it plays an essential role to life but, at the same time water resources are highly susceptible to changes and pressures imposed on the systems that comprise them, be that from climate change or from overconsumption by human activities^1^. Access to high-quality water is often local and intermittent, and constantly threatened by contamination and parochial interests. Organic chemicals are a growing body of anthropogenic contaminations that, upon release into the environment, are indeed breaking “nature’s social union”, that symbiosis between man and environment upon which the very life of man itself depends^2^. These substances may have different toxicological endpoints, namely, the killing of cells, the mutation of DNA in ways that may lead to cancer, and the disruption of chemical signalling mechanisms controlling cellular development, hence endocrine disruption^3–6^. Those substances with a verified impact on ecosystems and human health are the so-called contaminants of emerging concern (CECs), that represents a global sustainability concern^7,8^. In order to face this emerging water issue, it is becoming crucial to develop multiple effective treatments toward CECs. In the direction of an ideally perfect treatment we should walk on the route of a system simultaneously able to eliminate contaminants from the environments rather than filtering or absorbing them, (Degradative) effective toward the largest range of CECs possible (Versatile), effective at the natural pH of the matrix with a low energy and cost consumption (Viable)and able to work with a high turn-over number (TON; here intended as the ratio between concentration of degraded contaminant and the catalyst’s initial concentration) (Catalytic). In this direction advanced oxidation processes (AOPs) represent a promising solution^9–12^. Among AOPs ozonation in rapidly gaining ground in Europe, since it is able to effectively degrade a large range of organic contaminants and it is rather simple to mechanically implement^13,14^. However, the ozone (O_3_) generator is a sophisticated plant that requires a high energy consumption (around 7.2–12.3 kW/kg O_3_ generally and up to 132 kWh/m^3^ in terms of electrical energy consumption to remove 90% of contaminants in 1 m^3^ of a multi-contaminated water) imposing high capital and operative costs^14,15^. Therefore, ozone is a viable solution for large plants and rich jurisdictions, but it is not in less wealthy or smaller ones. Fenton reaction represents a viable alternative, since it employs cheap and environmentally-friendly starting reactants as iron ions (or other transition metal ions) and hydrogen peroxide (H_2_O_2_). The Fenton reaction generate hydroxyl radicals (^⋅^OH) allowing an effective, low-cost and versatile degradation of organic contaminants^16–19^. However, the process requires a pH adjustment to 3 and generates a large amount of sludge to dispose of, hence management efforts^19^. Commercial and cheap organic ligands (citric acid, Ethylenediamine-*N*,*N*ʹ-disuccinic acid (EDDS), Ethylenediaminetetraacetic acid (EDTA), and others) able to complex metal ions can overcome the latest limitations^20–23^. Indeed, the metal–ligand complexes can activate H_2_O_2_ driving an effective and versatile degradations of CECs at near-neutral pH avoiding the precipitation of the central ion, hence the sludge production^24,25^. Nevertheless, the reaction mechanism of these systems is still debated, and it is still not clear if these commercial ligands are able to induce a catalytic cycle or the metallo-organic complex works just as a H_2_O_2_ activator, hence a reagent^18,19^. Also, the process is limited by the stability of complex, largely lower than the corresponding oxidative systems employed in biological systems and last, the TON of these systems is generally low^26,27^. The brightest example of a metallo-organic catalyst inspired by biological systems was reported by Collins and co-workers, the so-called Fe-tertraamido macrocyclic ligand (Fe-TAML) series^28,29^. This catalysts can effectively lead the oxidative degradation of CECs with a high TON by means of highly stable complexes^30–32^. The limitation of TAMLs is their lower ability to perform at near-neutral pH, but at basic pH (10–11), limiting the applicability and increasing operative costs, also due to the high starting cost of the catalyst^32,33^. By time, the community, was able to develop a second generation of TAMLs. The latter exchanges a higher efficiency at near-neutral pH with a higher synthetic complexity^34,35^. Nevertheless, the more complex is the synthesis the higher is the cost of the catalyst, that should be kept as low as possible to pave the route of a practical implementation.

In the direction of a viable catalyst we learn from literature the existence of bioinspired metallo-organic structures employed in the field of organic synthesis for the selective functionalization of organic molecules^26^. Some of these structures are easier to synthesize, hence supposedly cheaper than TAMLs.

In this study three of these structures were selected and synthesized, hence employed in the field of water treatment as hydrogen peroxide activator. The study aims to assess the ability of easy-to-use bioinspired structure to activate H_2_O_2_ toward CECs degradation and to investigate their mode of action in water. This assessment seeks to be a starting platform to allow a further comparison with similar tools in the direction of an ideal degradative, versatile, viable, and catalytic (with high TON) treatment of CECs in water. In biological systems are already present structures acting in this fashion. Therefore, our work is inspired by the mechanism of biological oxidations to carry out water treatment.

Trispyridylamine (TPA), *N*,*N*ʹ-dimethyl-*N*,*N*ʹ-bis(2-pyridylmethyl)ethylenediamine (Me) and *N*,*N*ʹ-dimethyl-*N*,*N*ʹ-bis(2-pyridylmethyl)cyclohexane-trans-1,2-diamine (Mecy) were selected as organic ligands to complex iron and manganese ions, hence the two most present transition metals on the earth crust^36^. These three ligands also allow to evaluate the steric contribution of the complex in the reaction path, since the rigidity of the complexes increases from TPA to Me to Mecy^37^. The work evaluates the best oxidative conditions and the performance of the complexes toward several CECs and organics, namely: Phenol (PhOH), Estrone (E1), Triclosan (TCS), Oxybenzone (OXY), Diclofenac (DCF), Carbamazepine (CBZ), Erythromycin (ERY), Aspartame (ASP), Acesulfame K (ACE), Anisole (ANS) and 2,4-Dinitrotoluene (DNT). The contaminants were categorized as much as possible according to their molecular similarities rather than their usage destination. Along with the evaluation of the applicability of these systems in water, the study presents insights into the mode of actions of the complexes and as last stance it proposes the active species involved during the degradations in charge of each complex.

## Materials and methods

### Chemicals

E1, TCS, OXY, DCF, CBZ, ERY, ACE, 2-picolylamine, 2-(chloromethyl)-6-methylpyridine hydrochloride were purchased from Tokyo Chemical Industry (TCI). All the other reagents were purchased from Sigma-Aldrich. Water was of Milli-Q quality (TOC 2 ppb, resistivity 8.4 MΩ cm).

### Ligands synthesis

**Figure 1:**
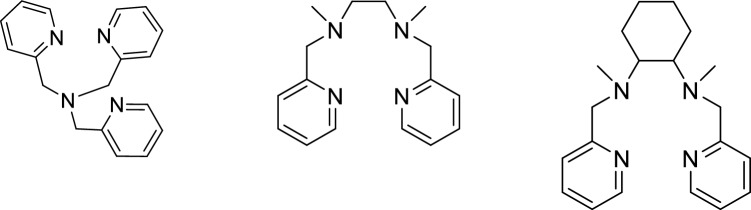
From left to right, the molecular structure of TPA, Me and Mecy.

TPA, Me and Mecy were synthesized in one step according to reported literature and their molecular structure are depicted in Fig. 1^38–40^.

### Complexes synthesis

Me-Mn(II), Me-Fe(II), Mecy-Fe(II), TPA-Fe(III) were synthesized according to a reported procedure^38,41,42^. The synthesis of Mecy-Mn(II) and TPA-Mn(II) were adapted from a reported procedure^38,43^. The synthetic procedures follow.

#### Me-Fe(II)

In Schlenk tubes on a vacuum line, Me (0.727 g, 2.69 mmol) in Ethanol (EtOH) (12 mL) was added dropwise to a solution of FeCl_2_.2H_2_O (0.438 g, 2.68 mmol) in EtOH (12 mL). The solution was stirred for 1 h, filtered, and washed with diethyl ether to yield a yellow powder (0.992 g, 2.5 mmol, 93%) (see Figs. S1, S2 in Supplementary Information (SI) for characterization).

#### Mecy-Fe(II)

In Schlenk tubes on a vacuum line, Mecy (1 g, 3.08 mmol) in EtOH (13 mL) was added dropwise to a solution of FeCl_2_.2H_2_O (0.502 g, 3.07 mmol) in EtOH (13 mL). The solution was stirred for 1 h, filtered, and washed with diethyl ether to yield a yellow powder (0.700 g, 1.55 mmol, 50%) (see Figs. S3, S4 in SI for characterization).

#### Me-Mn(II)

In a glovebox, Me (0.776 g, 2.87 mmol) in Methanol (MeOH) (1 mL) was added dropwise to a solution of MnCl_2_⋅4H_2_O (0.568 g, 2.86 mmol) in MeOH (2 mL). Halfway through the addition, a precipitate started to appear. The suspension was stirred for 30 min, filtered, and washed with diethyl ether to yield a white powder (0.961 g, 2.42 mmol, 84%) (see Figs. S5, S6 in SI for characterization).

#### Mecy-Mn(II)

In a glovebox, Mecy (0.729 g, 2.24 mmol) in MeOH (1 mL) was added dropwise to a solution of MnCl_2_.4H_2_O (0.445 g, 2.24 mmol) in MeOH (2 mL). Halfway through the addition, a precipitate started to appear. The suspension was stirred for 30 min, filtered, and washed with diethyl ether to yield a white powder (0.817 g, 1.81 mmol, 81%) (see Figs. S7, S8 in SI for characterization).

#### TPA-Fe(III)

In Schlenk tube under N_2_, TPA (0.3 g, 1.03 mmol) in the minimum amount of acetonitrile (MeCN) was added dropwise to a solution of FeCl_3_ (0.162 g, 1.03 mmol) in the minimum amount of MeCN. The solution was stirred for 30 min, filtered, and washed with diethyl ether to yield a yellow powder (0.374 g, 0.90 mmol, 90%) (see Fig. S9a in SI for characterization).

#### TPA-Fe(II)

The complex was prepared in-situ in water in a stock solution of 0.01 M dosing an equimolar ratio of TPA and FeCl_2_⋅2H_2_O. Aliquots of this stock solution were taken to perform the oxidation reactions (see Fig. S9b in SI for characterization).

#### TPA-Mn(II)

In Schlenk tube under N_2_, TPA (0.05 g, 0.17 mmol) in the minimum amount of MeOH was added dropwise to a solution of MnCl_2_⋅4H_2_O (0.27 g, 0.17 mmol) in the minimum amount of MeOH. Halfway through the addition, a precipitate started to appear. The solution was stirred for 2 h. Recrystallization from MeOH yielded to white crystals (0.374 g, 0.90 mmol, 90%) (see Fig. S9c in SI for characterization).

### Reaction conditions

The degradation experiments were carried out in homogeneous phase at room temperature, under continuous stirring, for 20 min, unless otherwise stated. Reactions were tested at different pH values (ranging from 5 to 8), in 10 mL of 10 mM phosphate buffer solution (PBS) or adjusting the pH with NaOH 0.1 M. Unless otherwise stated, the default initial concentrations in the experiments were as follows: 0.1 mM of complex, 0.1 mM of contaminant, and 4 stepwise additions of H_2_O_2_ corresponding each to a 0.1 mM concentration in the reaction system were made every 5 min. The rationale for stepwise reagent addition is that a too high concentration of H_2_O_2_ will consume the oxidant by disproportionation (catalase effect) rather than using it for degradation of the contaminant. Estrone was dissolved in pure iso-Propanol (i-PrOH) and dosed at 0.025 mM in water, due to its low solubility in water. Its degradation was tested just with manganese complexes since their oxidative ability is not affected in a solution of i-PrOH 0.025 mM (see below in the text). The catalytic efficiency expressed by TON was computed on the results of PhOH degradation with a complex:PhOH ratio 1 to 10.

### Analytical methods

UV–Vis spectrophotometric measurements were performed using a UV-2401-PC (Shimadzu). Cyclic Voltammetry (CV) experiments were performed using an Autolab potentiostat and a conventional 3 electrode device (C working electrode, Ag/AgCl reference electrode, Pt counter electrode). The electrolyte salt (TBAPF_6_) was recrystallized and all the glassware was dried at 110 °C before use. All the cyclic voltammograms (CVs) were recorded under argon in acetonitrile solution containing 0.1 M Bu_4_NPF_6_ at a scan rate of 0.1 V/s at 20 °C. All potential values are referred to Ag/AgCl. The CV characterization allow to define the redox potential of the complexes, hence their potential ability to work as catalysts during an oxidoreduction reaction.

The concentrations of contaminants in solution were monitored by ultra-high-performance liquid chromatography, with UV–Vis detection (UHPLC-UV) and liquid chromatography with triple quadrupole mass detection (LC/MS/MS). The used UHPLC U3000 (Thermo-Fisher) was equipped with a Autosampler (injection volume 7 µL), a quaternary pump for low-pressure gradients, a column oven (set at 25 °C). The column was a Kinetex C18-100A (length 100 mm, diameter 2.1 mm—particle size 1.7 μm EVO). The used LCMS-8050 (Shimadzu) was equipped with a Autosampler (injection volume 1 µL), a quaternary pump for low-pressure gradients, a column oven (set at 40 °C). The column was a Kinetex C18-100A (length 100 mm, diameter 2.1 mm—particle size 1.7 μm EVO). Elution of contaminants with both UHPLC 3000 and LCMS-8050 was carried out in gradient mode, with a mixture of A = water + 0.1% formic acid, and B = acetonitrile + 0.1% formic acid, at a flow rate of 0.5 mL/min. The gradient was always the same, namely time (t) = 0 min 100% of A − 0% of B; t = 1.5 min 70% of A − 30% of B; t = 2.0 min 0% of A − 100% of B; t = 4.0 min 0% of A − 100% of B; t = 4.1 min 100% of A − 0% of B; t = 5.0 min 100% of A − 0% of B. The contaminants analysed by UHPLC 3000 were: PhOH (λ = 270 nm, where λ = detection wavelength); TCS (λ = 282 nm); OXY (λ = 288 nm); DCF (λ = 275 nm); ACE (λ = 226 nm); ANS (λ = 270 nm); DNT (λ = 232 nm). On the other hand, E1, CBZ, ERY, ASP were analyzed by LCMS-8050.

### Electron paramagnetic resonance measurements

The spin-trap technique revealed the presence of radical species using a X-band Bruker-EMX spectrometer, equipped with a cylindrical cavity operating at 100 kHz field modulation. Experimental parameters were as follows: microwave frequency 9.86 GHz; microwave power 19.97 mW; modulation amplitude 2 Gauss; conversion time 15.43 ms. Spin-traps, 5,5-dimethyl-1-pyrroline-*N*-oxide (DMPO) or 2,2,6,6-tetramethylpiperidine (TEMP), each 17 mM, were added (separately in different experiments) to the other reagents at *t* = 0^44^.

## Results and discussion

### Understanding reaction behaviour

**Figure 2:**
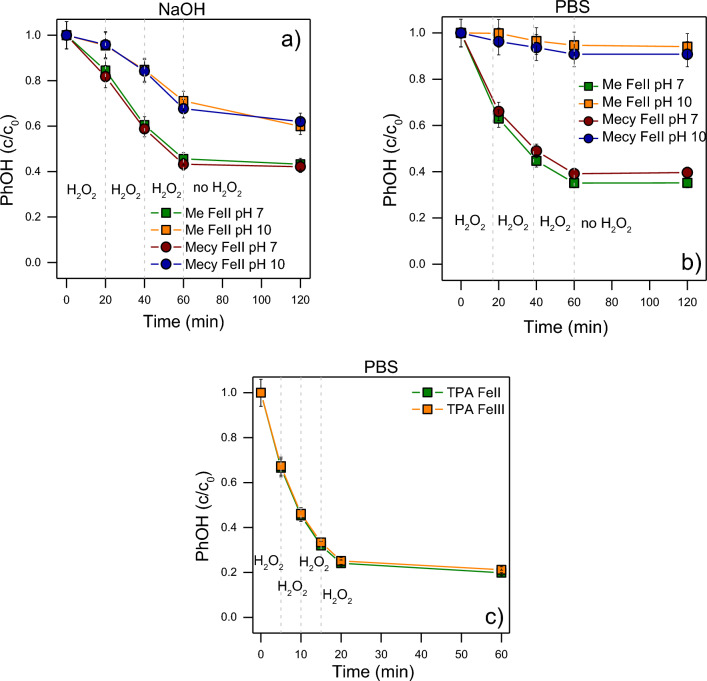
PhOH degradation at different conditions. Me-Fe(II) and Mecy-Fe(II) 0.1 mM were employed in the degradation of 0.1 mM of PhOH at different pH (7 and 10) adjusted with NaOH 0.1 mM (**a**) or in PBS 10 mM (**b**). H_2_O_2_ was dosed through 3 stepwise additions of 0.1 mM each 20 min. (**c**) TPA-Fe(II) and TPA-Fe(III) 0.1 mM were employed in the degradation of 0.1 mM of PhOH at pH 7 in PBS 10 mM. H_2_O_2_ was dosed through 4 stepwise additions of 0.1 mM, each 5 min.

Figure 2 shows a preliminary evaluation of the influence of time, buffer and amount of H_2_O_2_ on the degradation of PhOH. Figure 2a presents the efficiency of the oxidations in presence of the Me-Fe(II) and Mecy-Fe(II) at pH 7 and 10 with 3 additions of H_2_O_2_. The reaction performs better at pH 7 rather than 10, hence around the natural pH of a water matrix which ranges from 6.5 to 8.5. Also, the reaction proceeds in the first instants after the addition of H_2_O_2_. Indeed, if no H_2_O_2_ is added in the reaction environment, the oxydation does not proceed even over 60 min. It was also noticed that pH drops during the reaction (from 7 to 5 and from 10 to 7.5). This is possibly due to the formation of organic acids in the degradation pathway as reported in literature^30^. A buffer solution may help to better control the pH and its influence. Figure 2b shows that a PBS 10 mM aids to increase the degradation efficiency at pH 7, but it decreases at pH 10. These results highlight the crucial role of pH and its stability, corroborating the higher efficiency at neutral pH. Finally, the viability of the previously observed conditions was also tested by means of TPA-Fe(II, III) (Fig. 2c). Figure 2c demonstrates that the reaction can still efficiently occur by reducing the interval of H_2_O_2_ additions from 20 to 5 min, and that it is possible to reach higher degradation efficiency with a further addition of H_2_O_2_. Also, Fig. 2c did not show any clear influence of Fe(II) or Fe(III) on the efficiency of the reaction. This behaviour generates the hypothesis of a cyclic exchange of the 2 oxidation states during the reaction. Since Fe(III) is cheaper and more stable than Fe(II) over time, the following experiments were performed using TPA-Fe(III) complex.

Furthermore, the stability of the complexes in water was evaluated, aiming to observe the eventual release of the central ion in solution. Figure S10a–c in SI confirms the stability of the complexes in water during the reaction time. However, the eventual slight release of the central ion in solution will not compete with the degradations at pH higher than 5 (see Fig. S10d).

**Figure 3:**
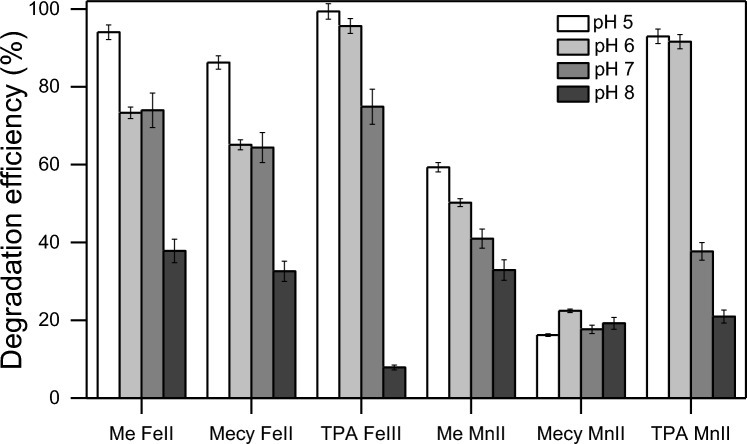
Degradation percentage of PhOH at different pH. The reactions were performed in 20 min in presence of PBS 10 mM, PhOH 0.1 mM, complexes (Me-Fe(II); Mecy-Fe(II); TPA-Fe(III); Me-Mn(II); Mecy-Mn(II); TPA-Mn(II)) 0.1 mM. H_2_O_2_ was dosed through 4 stepwise additions of 0.1 mM, each 5 min.

Figure 3 clearly shows the decrease of the reaction efficiency from pH 5 to 8 in presence of all the complexes tested with both iron and manganese ions. The lower reaction efficiency at higher pH is possibly due to the formation of µ-oxo dimers between the complexes, that can scavenge their role in the reaction and it is promoted at higher pH^37,45,46^. The following degradation experiments were performed in PBS 10 mM at pH 6 since it represents the optimal balance between the proximity to the natural pH of a water matrix and the efficiency of degradation. Also, H_2_O_2_ was dosed with 4 stepwise additions each 5 min according to the conditions found in Fig. 2.

### Complexes performance toward CECs

**Figure 4:**
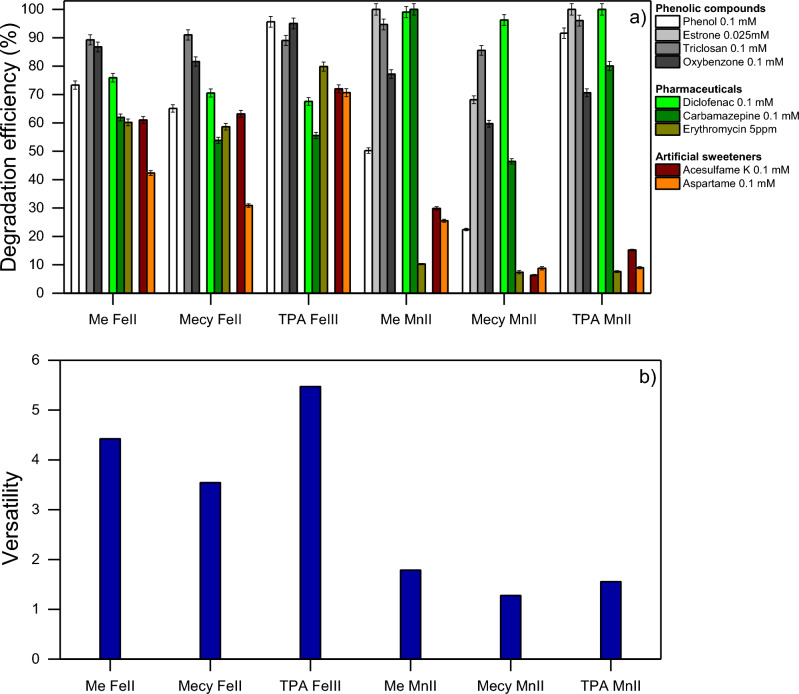
(**a**) Degradation percentage of the studied CECs. The reactions were performed at pH 6 in 20 min in presence of PBS 10 mM, Contaminant 0.1 mM, complexes (Me-Fe(II); Mecy-Fe(II); TPA-Fe(III); Me-Mn(II); Mecy-Mn(II); TPA-Mn(II)) 0.1 mM. H_2_O_2_ was dosed through 4 stepwise additions of 0.1 mM, each 5 min. (**b**) The versatility of the complexes tested toward CECs of (**a**).

Figure 4 reports the ability to induce the degradation of relevant micropollutants of each complex synthesized in this work namely, Me-Fe(II), Mecy-Fe(II), TPA-Fe(III), Me-Mn(II), Mecy-Mn(II), TPA-Mn(II). The micropollutants were selected according to their impact on human health and ecosystems and their presence in the environment (see Fig. S11 in SI for the molecular structure of the micropollutants). Phenolic compounds are widely spread in surface waters and owing to their molecular structure they mimic oestrogens and other hormones, hence they often act as endocrine disruptors^47–49^. Despite these compounds are easily naturally degraded, their rapid accumulation in the environment maintains their impact relevant. DCF and CBZ are generally persistent in the environment and affects embryos development and immune systems^50,51^. Antibiotics as ERY may cause antibiotics resistance if over accumulated into the environments^2,52^. Artificial sweeteners are posing a new threat to the environment since a relevant amount of them is resistant to human metabolism and traditional wastewater treatment plants (WWTP)^53,54^. Even if their ecotoxicological impact is still unknown, their suspected negative impact and their uncontrolled accumulation in the environment is a growing concern of the community.

Figure 4 clearly shows a higher degradation efficiency toward phenolic compounds in all the cases, while a generally lower toward ERY and artificial sweeteners. The systems with iron ions show a rather high efficiency toward all the contaminants tested (ranging from 30 to 95%), while the systems with manganese ions were proved more selective toward phenolic compounds, DCF and CBZ (from 50 to 99%) and showed a way lower efficiency toward ERY and artificial sweeteners (from 7 to 30%). Overall, the complexes with TPA were slightly more efficient compared to the corresponding complexes with the same central ion, while complexes with Mecy resulted less efficient. These results raised the hypothesis that the central ions play a more important role in the behaviour of the reaction than the ligands. Furthermore, the ligands do not perform according to their rigidity. Interestingly, the complexes with iron ions showed a higher versatility, here intended as the ability to efficiently degrade different contaminants without discrimination (see Eq. (E1) in SI for the formal definition of versatility), while the manganese complexes show a higher ability to discriminate among different contaminants (inter-selectivity). Specifically, the molecules with a higher density of electro-withdrawing groups (EWG) (as sulfonic, carboxylic, carbonylic, amidic, ester and nitric groups) are less susceptible toward oxidation. Among the CECs tested in this work ERY and artificial sweeteners lie in this ensemble and resulted the most recalcitrant contaminants in all the cases. The aforementioned behaviour of advanced oxidation was previously noticed in literature^31,34^. A selection of a wide range of both electron-rich and electron-poor contaminants helps the study to better highlight the effective treating power of the new tool (bioinspired complexes) and its versatility. Indeed, if highly susceptible compounds toward oxidation, as phenolic compounds, were selected, the study would communicate a biased super-high versatility of the tool. This distinct behaviour of the two series of complexes (with iron and manganese ions, respectively) arises from the possible homogeneous mode of action of each series depending mostly on the nature of the central ion rather than on the molecular structure of the ligand.

The parameter of versatility here presented does not aim to be an absolute parameter, it should be considered viable just in the framework of this communication. Further and specific studies may help to generalize this parameter.

### Investigation into the mode of action of the complexes

Previous data demonstrated the ability of this new tool to effectively perform toward the degradation of micropollutants, even toward the most recalcitrant as the artificial sweeteners. However, it is still not clear how these complexes act in the reaction environment and which are the active species involved.

**Figure 5:**
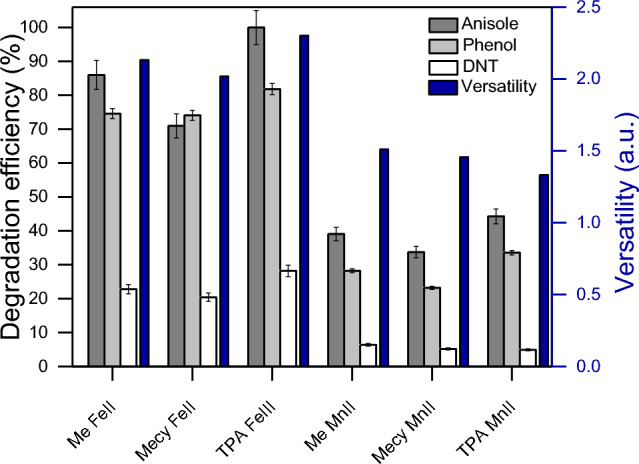
Degradation percentage of different organic contaminants in the same water matrix. The reactions were performed at pH 6 in 20 min in presence of PBS 10 mM, each contaminant 0.02 mM, complexes (Me-Fe(II); Mecy-Fe(II); TPA-Fe(III); Me-Mn(II); Mecy-Mn(II); TPA-Mn(II)) 0.1 mM. H_2_O_2_ was dosed through 4 stepwise additions of 0.1 mM, each 5 min. On the right y axis in blue the versatility of the systems tested.

The experiment of Fig. 5 aims to better understand the nature of the active species involved in the degradation reactions. In this regard, an artificial wastewater with an equimolar amount (0.02 mM) of a homologous series of three different compounds was produced, where ANS and PhOH are more electron-rich and DNT is less due to the higher presence of electron donor groups (EDG) and EWG respectively (see Fig. S11 in SI for the molecular structure). The results of Fig. 5 show higher efficiency toward the more electron-rich substrates in all the cases. Phenolic compounds may affect the efficiency of the reaction by interacting with the central ion of the complexes, as reported in previous literature in case of organic solvents^55^. However, no interaction with PhOH or catechol and the central ion (iron or manganese ions) was observed in water (see Fig. S12 in SI). This is most likely owing to the stronger solvent cage of water compared to polar organic solvents as MeCN, that thwart the interaction PhOH-Fe(Mn). This evidence is corroborated by an even higher percentage of degradation of ANS which is as polar as PhOH, but due to the presence of the methyl group on oxygen it has a steric hindrance that prevent any interaction with the central ions of the complexes. Therefore, this new tool shows a tendence toward electron-rich molecules as also generally observed in Fig. 4a.

Figure 5 interestingly shows the distinct behaviour, hence the relative versatility, between iron and manganese complexes is maintained as in Fig. 4b even changing the series of organics. Therefore, it is reasonable to hypothesize two different modes of action between the two series of complexes with iron and manganese. Also, the versatility can be considered strongly related to these modes of action rather than to the molecular structure of the ligands.

**Figure 6:**
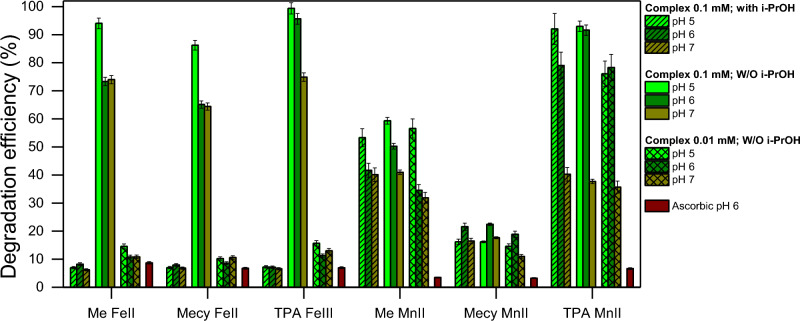
Effects of different conditions on PhOH degradation. i-PrOH 0.025 mM (slant bars); Complex 0.1 mM (empty bars); Complex 0.01 mM (crossed-lines bars); Ascorbic acid 0.025 mM (red bars). The reactions were performed at pH 6 in 20 min in presence of PBS 10 mM, Contaminant 0.1 mM, H_2_O_2_ was dosed through 4 stepwise additions of 0.1 mM, each 5 min.

Figure 6 corroborates the hypothesis of the two different modes of action among the two series of complexes. Indeed, the reaction with iron complexes is scavenged by i-PrOH and by a 10 times lower amount of the complex at all the pH tested (5, 6, 7), while the manganese complexes are not affected by these reaction conditions. The series of manganese complexes acts with a more distinct catalytic behaviour and a higher TON, intended as the ratio between the efficiency of the degradation and the starting complex concentration (PhOH in case of Fig. 6). Also, i-PrOH is a well known ^⋅^OH scavenger, thus the latter may be the predominant species in the systems with iron complexes while another oxidative species is involved in the systems with manganese. All this induces the hypothesis of two active species involved in the two systems with iron and manganese. Also, the results further show that is the central ion which plays a crucial role in the mode of action of the system rather than the molecular structure of the ligands that only slightly influences the efficiency of degradation. Furthermore, Fig. 6 shows the ability of ascorbic acid at pH 6 to quench all the reactions, hence the active species.

**Figure 7:**
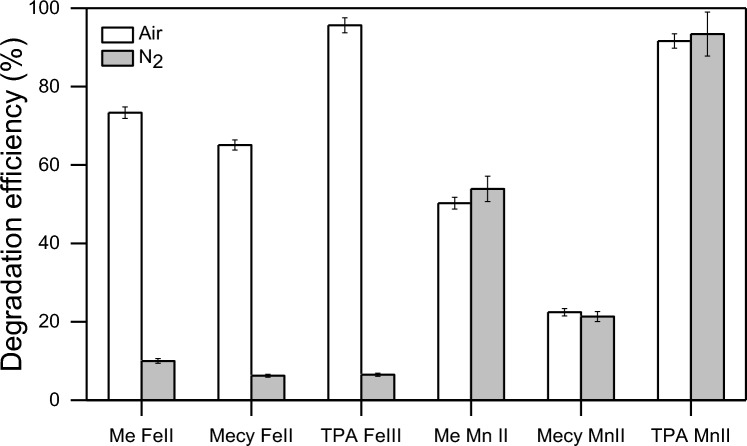
Effect of dioxygen (air) and inert atmosphere (N_2_) on PhOH degradation. Ascorbic acid was used as reaction quencher in these experiments. The reactions were performed in 20 min in presence of PBS 10 mM, PhOH 0.1 mM, complexes (Me-Fe(II); Mecy-Fe(II); TPA-Fe(III); Me-Mn(II); Mecy-Mn(II); TPA-Mn(II)) 0.1 mM. H_2_O_2_ was dosed through 4 stepwise additions of 0.1 mM, each 5 min. The reactions with iron systems are scavenged in absence of dioxygen.

Literature reports that dioxygen can deeply affect the reaction efficiency when ^⋅^OH is the predominant active species^56–58^. With this respect, the experiments of Fig. 7 aim to further corroborate the eventual predominant presence of ^⋅^OH in the systems with iron complexes. Indeed, the results demonstrate the importance of dioxygen on the progress of the reactions in iron systems that conversely do not act in inert atmosphere (see Fig. 7). On the other hand, the systems with manganese complexes are not affected by this change in the same fashion of Fig. 6. This study has now presented multiple proof to communicate a distinct behaviour of the systems with iron and manganese and their relative mode of action. However, a direct observation of the active species in Mn systems is still lacking.

### Observations the predominant active species

**Figure 8:**
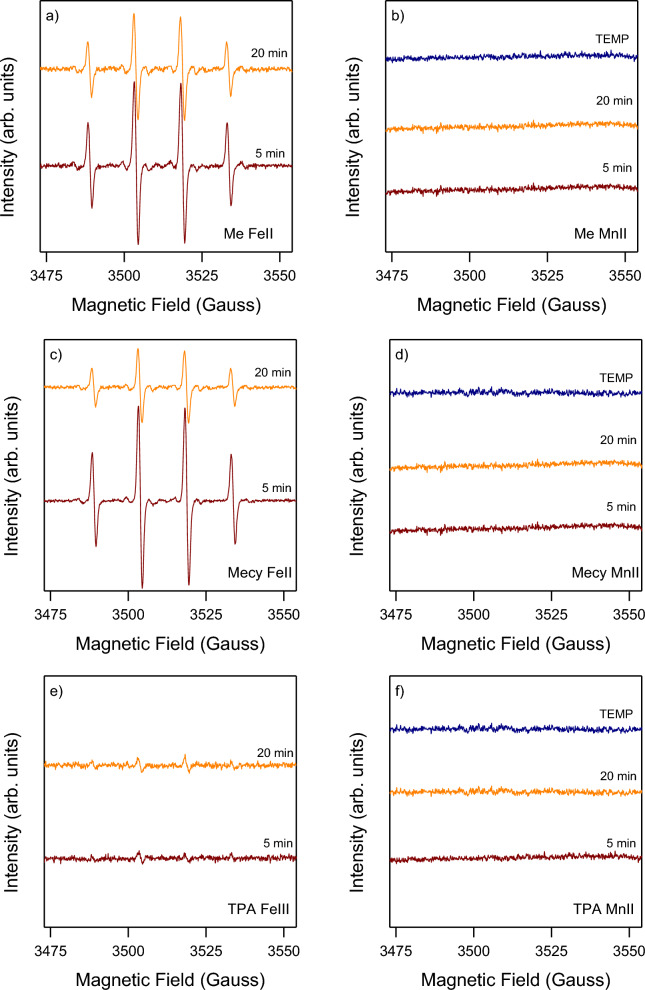
EPR spectrum of the radical transient species during the oxydations. Follow the experimental conditions of each graph: (**a**) Me-Fe(II) 0.1 mM, PhOH 0.1 mM, H_2_O_2_ 0.4 mM; (**b**) Me-Mn(II) 0.1 mM, PhOH 0.1 mM, H_2_O_2_ 0.4 mM; (**c**) Mecy-Fe(II) 0.1 mM, PhOH 0.1 mM, H_2_O_2_ 0.4 mM; (**d**) Mecy-Mn(II) 0.1 mM, PhOH 0.1 mM, H_2_O_2_ 0.4 mM; (**e**) TPA-Fe(III) 0.1 mM, PhOH 0.1 mM, H_2_O_2_ 0.4 mM; (**f**) TPA-Mn(II) 0.1 mM, PhOH 0.1 mM, H_2_O_2_ 0.4 mM. H_2_O_2_ was added at once in all the reactions. DMPO 17 mM was used as spin trap in (**a–f**), while in (**b,d,f**) the reaction was repeated in presence of TEMP 17 mM as spin trap in place of DMPO and the spectrum taken at 5 min. The results suggest that the active species is ^⋅^OH in (**a,c,e**) and a non-radical one in (**b,d,f**).

EPR measurements allowed to get insight into the active species involved. Figure 8 showed again a distinct behaviour between iron and manganese complexes (see Fig. 8a,c,e against Fig. 8b,d,f). The reactions were carried out in the presence of a spin trap to detect potential radical species released in solution and analysed by EPR. Iron systems clearly show an adduct with DMPO, hence the presence of a radical species. The parameters of the simulation were as follows: a_N_ = 14.9 Gauss; a_H_ = 14.9 Gauss; g = 2.0057 (correlation coefficient = 0.9995), in agreement with literature data for the DMPO-^⋅^OH adduct, hence the detected radical species is ^⋅^OH^59^. In the iron systems the ^⋅^OH is immediately formed in all the systems and it slightly declines after 20 min with the exception of TPA-Fe(III) system where the presence of ^⋅^OH is poor from the beginning and it is kept constant or it is slightly increased over the reaction time (20 min). On the other hand, in manganese systems there is no observation of adducts with both DMPO and TEMP (selective for singlet oxygen)^56,60^. Therefore, the manganese systems follow a non-radical path. As reported in literature in the case of the same systems in organic solvents the alternative to a radical pathway is a metal-based active species (i.e. high-valent Mn-Oxo species), hence a species able to mimic the mechanism of biological systems^26,61^. This hypothesis would further demonstrate the catalytic behaviour of manganese systems (see Fig. 6), since a metal-based species is the necessary condition to act through a catalytic cycle.

### Overall view of the complexes’ performance

**Figure 9:**
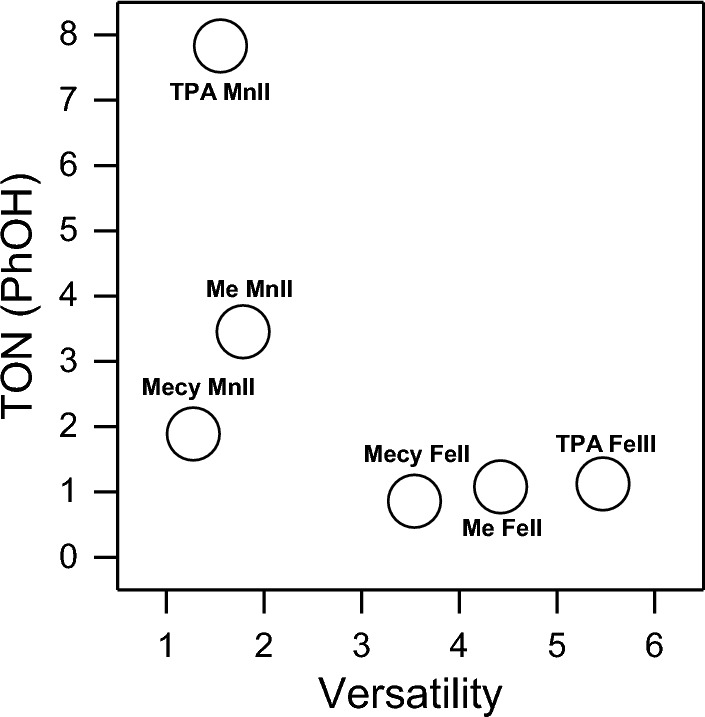
Relationship between versatility (computed on the degradation efficiencies for all the contaminants tested according to E1 in SI) and TON (computed on data of Fig. 6, hence complex 0.01 mM, PhOH 0.1 mM) for all the complexes tested in this study.

Figure 9 reports a summary of the relative efficiencies of the systems tested in this work. It schematically shows a poled behaviour between iron and manganese complexes. Indeed, the systems with iron are more versatile and conversely the manganese systems show a higher inter-selectivity. The manganese systems have a more distinct catalytic behaviour with a higher TON, while the iron systems follow a radical path, hence a lower TON. This stark distinct behaviour between the two systems allows proposing a versatile use of this new tool in a potential real application. Indeed, by simply changing the central metal it is possible to have either a complex able to degrade a plethora of contaminants in a multi-contaminated matrix (iron systems) or a complex able to specifically degrade target contaminants, likewise ensuring a higher TON (manganese systems). Figure 9 defined a trade-off between versatility and TON in the framework of the systems tested in this work. The TON-Versatility relationship can be use in future with the aim to define a system able to overcome the trade-off, hence with both high versatility and high TON. Overall, this work reports a slightly higher efficiency of TPA-Fe(III) compared to others and a lower efficiency of degradation of Mecy complexes. In order to rigorously rationalize all the degradation efficiency differences exhibited by the different complexes will be furtherly required (i) to determine their fate and identify possible self-degradation; (ii) to analyse the nature and amount of oxidation products.

## Conclusions

This work presented a series of easy-to synthesize bioinspired metal complexes used to promote advanced oxidations in water toward CECs. Overall, the following conclusions can be drawn:

i. All the ligands require one step synthesis.
ii. All the complexes showed an activity toward CECs.
iii. All the complexes proved a tendence toward electron-rich compounds.
iv. The central ion plays a crucial role to lead the mode of action of the reaction. Specifically, Mn(II) induces a non-radical pathway while iron ions induce a predominant radical one.
v. ^⋅^OH is the predominant radical species in iron systems, while was not isolated any specific active species in manganese systems.
vi. The active species has a predominant influence toward contaminants degradation regardless of the ligand’s molecular structure.
vii. Manganese systems are more selective (in terms of inter-selectivity), hence they are less versatile than iron systems, but they have a higher TON.
viii. No direct correlation between the rigidity of the complexes and their degradation efficiency was noticed.

Ozonation and Fenton process are consolidated technologies, and they are highly effective toward CECs degradation. However, ozone generation requires high costs and energy demand, and it is not easily implementable in small jurisdictions or poor ones. Fenton is a cheap treatment, but requires management efforts. TAMLs series conceptually represented a brilliant alternative to ozonation in rural areas and a solution to better control the Fenton process. However, its synthesis is complex and not easily scalable. Simpler structures are then required. The complexes here presented may pave the route of the development of similar structures, but simpler to synthesize and more performing in terms of versatility and catalytic efficiency.

### Supplementary Information

Supplementary Information.

## Data Availability

All data generated or analysed during this study are included in this published article and its Supplementary Information files.
