# Supplementary material for: Assessment of new hydrogen peroxide activators in water and comparison of their active species toward contaminants of emerging concern
Source: Sci Rep. 2024 Apr 23;14:9301. doi: 10.1038/s41598-024-59381-0 (PMC11039771; doi:10.1038/s41598-024-59381-0)
Supplement: Supplementary file 1 — Supplementary Information. [file 41598_2024_59381_MOESM1_ESM.docx]

**Supplementary Information**

**Assessment of new hydrogen peroxide activators in water and comparison of their active species toward contaminants of emerging concern**

### **Giulio Farinelli^1*^ ⸱ Jean-Noël Rebilly^2^ ⸱ Frédéric Banse^2^ ⸱ Marc Cretin^1^ ⸱ Damien Quemener^1**^**

^1^ *Institut Européen des Membranes, IEM-UMR 5635, Univ de Montpellier, ENSCM, CNRS, 34090, Montpellier, France*

^2^ *Institut de Chimie Moléculaire et des Matériaux d'Orsay (ICMMO), Université Paris-Saclay, CNRS, 91400 Orsay, France*

* **Corresponding author**: Dr. Giulio Farinelli, E-mail: [giulio.farinelli@umontpellier.fr](mailto:giulio.farinelli@umontpellier.fr)

** **Corresponding author**: Prof. Damien Quemener, E-mail: damien.quemener@umontpellier.fr

**Equation E1.** Formal definition of versatility.

$$Versatility=\frac{Average of the efficiency of CECs degradations}{St. Dev.of the efficiency of CECs degradations}$$


**Figure S1**. UV-vis spectrum (298 K, MeOH) of ME-Fe(II) (Concentration = 10^-4^ M).

**Figure S2**. CV of Me-Fe(II) (Concentration = 2 x 10^-3^ M) in MeCN (298 K). Workin Electrode (WE): Glassy Carbon (GC), Counter Electrode (CE): Pt, Ref: Ag/AgCl, NBu_4_PF_6_ 0.1 M. Full scale (a/) and zoom on the Fe^III^/Fe^II^ wave (b/). E_1/2_ (Fe^III^/Fe^II^)= 0.23 V (∆E = 100 mV).

**Figure S3**. UV-vis spectrum (298 K, MeOH) of Mecy-Fe(II) (Concentration = 10^-4^ M).

**Figure S4**. CV of Mecy-Fe(II) (Concentration = 2 x 10^-3^ M) in MeCN (298 K). WE: GC, CE: Pt, Ref: Ag/AgCl, NBu_4_PF_6_ 0.1 M. Full scale (a/) and zoom on the Fe^III^/Fe^II^ wave (b/). E_1/2_ (Fe^III^/Fe^II^)= 0.23 V (∆E = 200 mV).

**Figure S5**. UV-vis spectrum (298 K, MeOH) of Me-Mn(II) (Concentration = 10^-4^ M).

**Figure S6**. CV of Me-Mn(II) (Concentration = 2 x 10^-3^ M) in MeCN (298 K). WE: GC, CE: Pt, Ref: Ag/AgCl, NBu_4_PF_6_ 0.1 M. Full scale (a/) and zoom on the Mn^III^/Mn^II^ wave (b/). E_1/2_ (Mn^III^/Mn^II^)= 0.75 V (∆E = 100 mV).

**Figure S7**. UV-vis spectrum (298 K, MeOH) of Mecy-Mn(II) (Concentration = 10^-4^ M).

**Figure S8**. CV of Mecy-Mn(II) (Concentration = 2 x 10^-3^ M) in MeCN (298 K). WE: GC, CE: Pt, Ref: Ag/AgCl, NBu_4_PF_6_ 0.1 M. Full scale (a/) and zoom on the Mn^III^/Mn^II^ wave (b/). E_1/2_ (Mn^III^/Mn^II^)= 0.71 V (∆E = 100 mV).

|  |  |
| --- | --- |
|  | |

**Figure S9**. UV-vis spectrum (298 K, H_2_O) of TPA-Fe(III) (a), TPA-Fe(II) (b) and TPA-Mn(II) (c) (Concentration = 10^-4^ M).

|  |  |
| --- | --- |
|  |  |

**Figure S10**. UV-vis spectrum (298 K, H_2_O) of Me-Fe(II) (a); Mecy-Fe(II) (b); TPA-Fe(III) (c) (Concentration = 10^-4^ M) over time. d) PhOH degradation efficiency ranging from pH 5 to 7. The reactions were performed in 20 min in presence of PBS 10 mM, PhOH 0.1 mM, FeCl_2_ and MnCl_2_ 0.1 mM. H_2_O_2_ was dosed through 4 stepwise additions of 0.1 mM, each 5 minutes. The inactivity of the reaction is mostly due to the immediate precipitation of the metals in presence of PBS at pH higher than 3 as required by the traditional Fenton process in water.


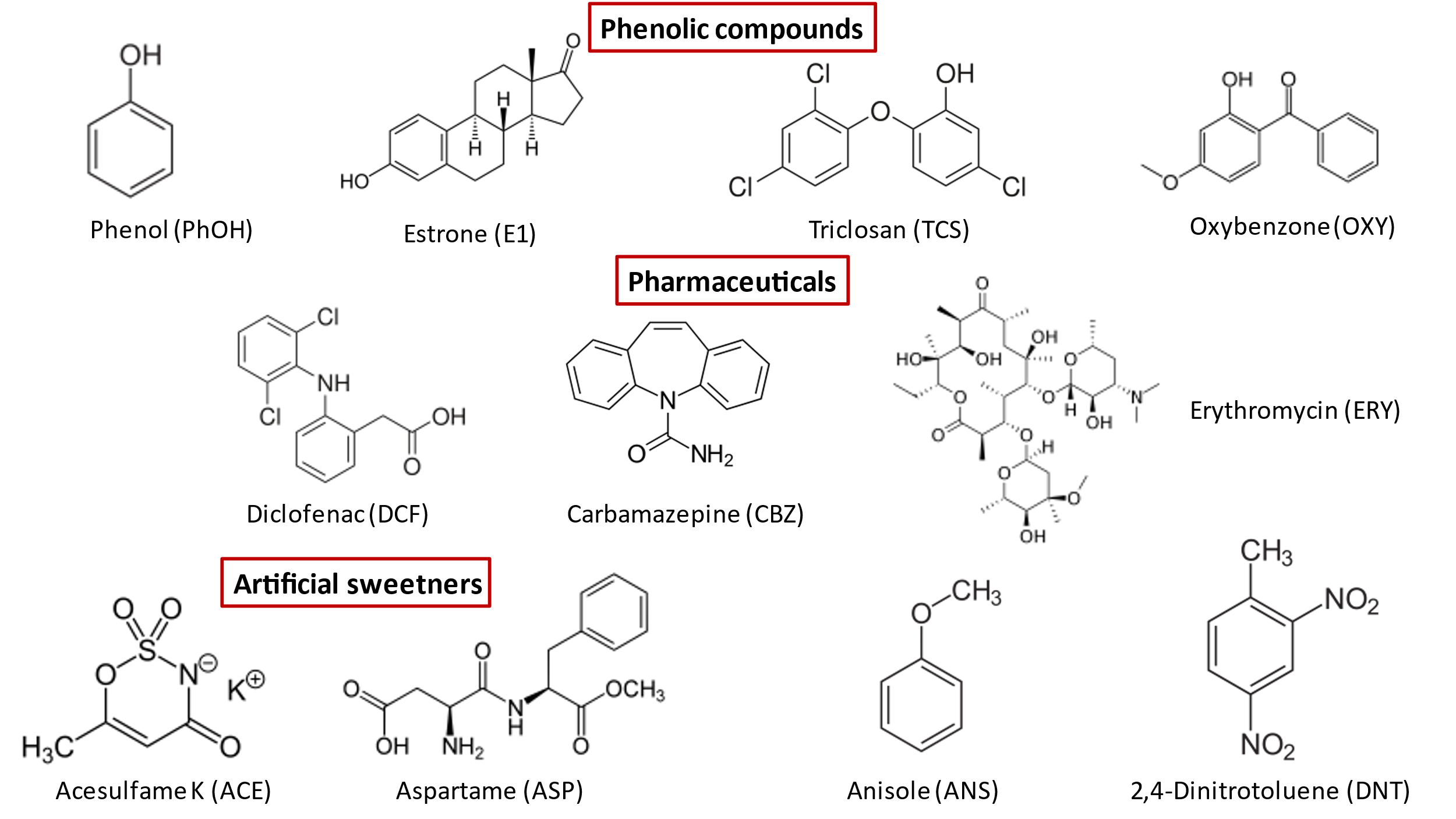


**Figure S11**. Molecular structures of organics tested in the present study.

|  |  |
| --- | --- |
|  |  |
|  |  |
|  |  |
|  |  |

**Figure S12**. UV-vis spectrum (298 K, H_2_O) of Me-Fe(II) (a); Me-Mn(II) (b) Mecy-Fe(II) (c); Mecy-Mn(II) (d); TPA-Fe(II) (e); TPA-Mn(II) (f) and PhOH and UV-vis spectrum (298 K, H_2_O) of Me-Fe(II) (g); Me-Mn(II) (h) Mecy-Fe(II) (i); Mecy-Mn(II) (j) and catechol (CatOH). The starting concentration of the complexes was 10^-4^ M. PhOH and CatOH were sequentially added in the same cuvette to reach 0.1 mM and 0.2 mM after. The band at 260 nm is related to the added bands of the organics and the complexes that absorb in the same range. Indeed, it increases with the concentration of the organics already dosed equimolarly with the complex. In all the spectrums there is no evidence of the formation of a new band, irrefutable proof of a new interaction. Therefore, the central ions (iron and manganese) do not interact with PhOH and CatOH in water.
